# Prevalence and Risk Factors Associated with Potentially Inappropriate Prescribing According to STOPP-2 Criteria among Discharged Older Patients—An Observational Retrospective Study

**DOI:** 10.3390/ph16060852

**Published:** 2023-06-07

**Authors:** Mariana Sipos, Andreea Farcas, Daniel Corneliu Leucuta, Noémi-Beátrix Bulik, Madalina Huruba, Dan Dumitrascu, Cristina Mogosan

**Affiliations:** 1 Department of Pharmacology, Physiology and Physiopathology, Faculty of Pharmacy, “Iuliu Haţieganu” University of Medicine and Pharmacy, 400010 Cluj-Napoca, Romania; mariana.sipos89@gmail.com (M.S.); cmogosan@umfcluj.ro (C.M.); 2 Pharmacovigilance Research Center, “Iuliu Hatieganu” University of Medicine and Pharmacy, 400349 Cluj-Napoca, Romania; 3 Department of Medical Informatics and Biostatistics, “Iuliu Hatieganu” University of Medicine and Pharmacy, 400012 Cluj-Napoca, Romania; dleucuta@umfcluj.ro; 4 Department of Internal Medicine, Medical Clinic 2, Faculty of Medicine, “Iuliu Hatieganu” University of Medicine and Pharmacy, 40000 Cluj-Napoca, Romania; ddumitrascu@umfcluj.ro

**Keywords:** potential inappropriate prescribing, elderly, STOPP-2, internal medicine

## Abstract

Pharmacokinetic and pharmacodynamic changes associated with old age, along with multimorbidity and polypharmacy might lead to inappropriate prescribing and adverse reactions. Explicit criteria such as the Screening tool of older people’s prescribing (STOPP) are useful to identify potential inappropriate prescribing’s (PIPs). Our retrospective study included discharge papers from patients aged ≥65 years, from an internal medicine department in Romania (January–June 2018). A subset of the STOPP-2 criteria was used to assess the prevalence and characteristics of PIPs. Regression analysis was performed to evaluate the impact of associated risk factors (i.e., age, gender, polypharmacy and specific disease). Out of the 516 discharge papers analyzed, 417 were further assessed for PIPs. Patients’ mean age was 75 years, 61.63% were female and 55.16% had at least one PIP, with 81.30% having one or two PIPs. Antithrombotic agents in patients with significant bleeding risk was the most prevalent PIP (23.98%), followed by the use of benzodiazepines (9.11%). Polypharmacy, extreme (>10 drugs) polypharmacy, hypertension and congestive heart failure were found as independent risk factors. PIP was prevalent and increased with (extreme) polypharmacy and specific cardiac disease. Comprehensive criteria like STOPP should be regularly used in clinical practice to identify PIPs to prevent potential harm.

## 1. Introduction

Based on the results of the 2021 census, the elderly population in Romania is continuously increasing, reaching 19.6% out of the total population [1]. Despite a similar trend in Europe [2], Romania remains the country with the second to last life expectancy in the European Union (EU) (74.2 years in Romania versus 80.6 years in the EU) [3].

Physiological changes and a progressive decline of the normal function of organs and systems are known to happen with old age [4]. Pharmacokinetic changes associated with old age are related to changes in body composition with an increase in body fat and a decrease in water content as well as a decrease in the renal and hepatic functions [5,6]. Decrease of total body water and increase in body fat affect drug distribution. Reduction of liver dimension and hepatic blood flow can reduce drug clearance or availability of drugs that undergo a significant first pass metabolism. Altered renal function affect the clearance of renal eliminated drugs (e.g., digoxin, diuretics), leading to a risk of toxicity for these drugs [5,6]. Pharmacodynamic changes, like a decrease in the number of synapses in the brain, leave the elderly with a higher sensitivity to drugs like benzodiazepines, anaesthetics and opioids [5]. 

Old age particularities, such as modified pharmacokinetics and pharmacodynamics, comorbidities and by consequence, polypharmacy [7,8,9,10] are considered risk factors for potentially inappropriate prescribing (PIP) [7,11]. Polypharmacy and PIP are known factors for adverse drug reactions (ADRs). The impact of PIP was evaluated across different studies, showing that PIP can lead to an increased number of hospitalization days and/or hospital visits, high mortality rates, and high medical costs [7,12,13,14]. 

Explicit criteria such as Beers’ Criteria, Medication Appropriateness Index and Screening Tool to Alert to Right Treatment (START)/Screening tool of older people’s prescribing (STOPP) are used to facilitate the medication review in older patients and identify PIP [15,16,17,18,19]. PIP as per START/STOPP criteria defines both potentially inappropriate medicines (PIM) identified by STOPP criteria or potential prescribing omissions identified by START criteria [15]. Aside these criteria, the Charlson Comorbidity Index (CCI) is a weighted score that checks for the presence of 19 diseases such as chronic renal failure, diabetes, stroke or cancer, used to evaluate the impact of co-morbid conditions and their impact on ten-year mortality [20,21]. 

For clinical practice, the benefit of STOPP criteria in reducing ADRs and improving medication appropriateness has been proven across numerous studies conducted in different settings such as institutionalized and hospitalized patients [22,23,24,25] or ambulatory setting [12,24], as well as in various countries such as UK [26], the Netherlands [27], Sweden [28], Korea [11] and others [12,22,24]. The reported prevalence of PIP varied between 20% and 85% [14,22,25,26,29], with high impact on both health [23,30] and economic aspects [25,28]. In Romania, studies evaluated PIP using different criteria, mostly in ambulatory setting (electronic prescriptions at pharmacy level) and in institutionalized older adults, with prevalence of PIMs varying from 26% [12] to 85% [24,31]. At discharge, 40% of patients have discrepancies in their treatments such as duplications, omissions or inappropriate prescribing, due to changes made in their therapy during hospitalization [32], and therefore there is a need to study this phenomenon in a hospital based setting. 

As such, the objectives of this study were to assess the prevalence of PIP in discharge medication of hospitalized patients, using selected criteria from STOPP version 2 (STOPP-2) [15], and to evaluate the potential impact of the associated factors (i.e., age, sex, comorbidities, polypharmacy and various diagnoses) on PIP. 

## 2. Results

### 2.1. Characteristics of Included Patients

A total of 516 charts of patients discharged between January and July 2018 were considered for the analysis. 39 were excluded due to fatal outcome or transfer to another hospital department, and 53 had two or three hospital admissions during the study period. Additionally, seven patients were excluded from the PIP analysis as they had none of the drugs included in the STOPP-2 criteria prescribed. 

A total of 417 patients were further analyzed, with an average age of 75.62, ranging between 65–96 years, and 61.63% were female. The mean number of diagnoses at discharge was 13.52 and hypertension was the most frequent (82.25%). Overall, 55.16% (230 patients) presented at least one PIP. An additional analysis was conducted to compare the PIP group versus the non-PIP group (Table 1). In the Overall group, 58.51% patients had a moderate CCI, between 2 and 4, with a similar distribution in the PIP versus non-PIP group. In comparison, 21.82% had a severe index (CCI ≥ 5), with a higher distribution in the PIP group (*p* = 0.006). We found a statistical significance (*p* < 0.05) when comparing patients with PIP versus those without, for CCI, hypertension, atrial fibrillation and congestive heart failure, indicating that patients with these conditions or a higher CCI are more predisposed to PIP.

### 2.2. Polypharmacy and Prescribed Drugs

Overall polypharmacy was identified in 86.33% of patients, with a higher prevalence in patients with PIP (93.48%). Extreme polypharmacy was identified in 26.62% of the Overall group, and in 34.78% patients with PIPs. The median number of chronic prescribed drugs, used for the assessment of polypharmacy was of 8, with a higher median value in the PIP group (8.5 drugs) versus non-PIP group (6 drugs).

Overall, antithrombotic agents, generally represented by the ATC group B01AC—platelet aggregation inhibitors excluding heparin, were the most prescribed, 75.06% of patients were prescribed at least a representative of this drug class. Antithrombotic drugs were prescribed more in the PIP group (82.61%) compared to the non-PIP group (67.21%). Beta blocking agents, mostly represented by selective beta blockers, were the second most prescribed drugs (66.19%), followed by lipid modifying agents (53.24%). Agents acting on the renin–angiotensin system were also prescribed to 51.56% of patients in the Overall group, with almost 60% of patients in the PIP group having been prescribed a drug from this class as compared to 43% in the non-PIP group (Table 1). 

### 2.3. Potentially Inappropriate Prescribing and Associated Risk Factors

**Figure 1:**
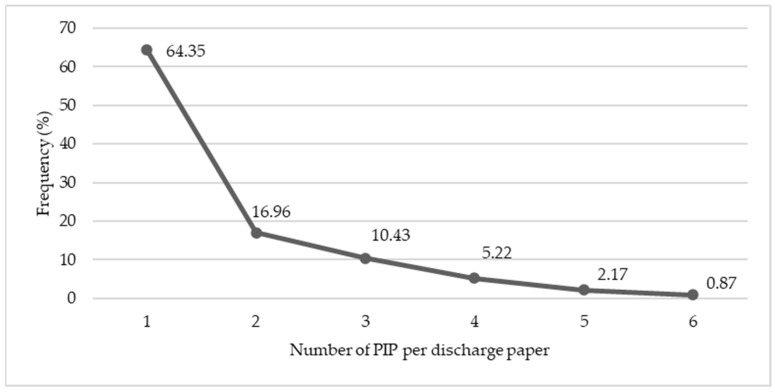
Distribution of PIP per discharge paper in the PIP group. PIP, potential inappropriate prescribing.

Overall, a total of 383 PIPs were identified in 230 patients (55.16%). The number of PIPs per patient varied between one and six, with 81.30% having one or two PIPs, 15.65% with three or four PIPs, and 3.04% with more than five PIPs (Figure 1). 

**Figure 2:**
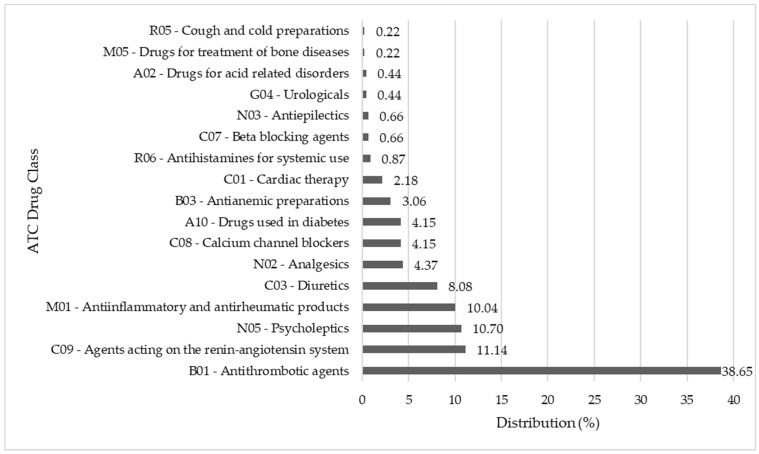
Distribution of drugs involved in PIP. ATC, Anatomical Therapeutics Chemical classification system; PIP, potential inappropriate prescribing.

We found that the drugs most frequently involved in PIP were *Antithrombotic Agents* (*B01A*)—identified in 38.65% of the detected PIP, followed by *Agents acting on Renin-Angiotensin system* (*C09;* Angiotensin-converting-enzyme inhibitors [ACEi], *C09A*, Angiotensin II receptors plain [*C09C*] and in combination [*C09D*])—in 11.14%, *Psycholeptics* (*N05;* Anxiolytics [*N05B*], Hypnotics and sedatives [N05C])—in 10.70% and *Antiinflammatory and antirheumatic products, non-steroids* (*M01A*)—in 10.04%. The other drugs involved in PIP had a distribution under 10% (Figure 2).

The most common STOPP-2 criteria were related to the cardiac system, and associated with the prescription of antithrombotic agents. 23.98% of patients with PIP had antithrombotic agents with concurrent significant bleeding risk, while 4.56% had aspirin prescribed in combination with vitamin K antagonists, direct thrombin inhibitors or factor Xa inhibitors in patients with chronic atrial fibrillation. 9.11% of patients had PIP associated with prescribing benzodiazepines. 7.67% of patients were prescribed aldosterone antagonists with concurrent potassium-conserving drugs without a clear recommendation for serum potassium monitoring, while 3.84% were prescribed ACEi with existing hyperkalemia. 4.32% patients had duplicate class prescriptions associated with the prescription of furosemide, acetaminophen or ACEi prescribed along with angiotensin receptor blockers (Table 2). The list with all STOPP-2 criteria found in our study is in Appendix A. 

### 2.4. Regression Analysis 

We performed a regression analysis having PIP as the dependent variable including independent variables for which a positive link was found in the univariate analysis. Moreover, we entered into the multivariate model age, gender and hospitalization days as possible cofounders even though they were not statistically significant in the univariate analysis. Although a positive link was found between the number of hospitalization days, number of diagnoses at discharge, CCI and the number of prescribed drugs in the univariate analysis, the regression analysis showed that only polypharmacy and extreme polypharmacy were an independent risk factor for PIP (*p* < 0.05). Hypertension and congestive heart failure were also positively correlated with PIP. These two diagnoses were identified as well as independent risk factors for PIP in the regression analysis (*p* < 0.05) performed in the study group (Table 3).

## 3. Discussion

### 3.1. Polypharmacy and Comorbidities

Polypharmacy was reported for over 80% and the median number of drugs was 8 in the Overall group. A higher prevalence, statistically significant (*p* < 0.001), was found in the PIP group, where the median number of drugs was 8.5 and the prevalence of polypharmacy was 93.48%, compared to the non-PIP group with a median of 6 drugs and a prevalence of 77.54%. Similarly, Cabello et al., in a study on 275 patients from an internal medicine department, reported a higher median number of drugs prescribed in the PIP group (11 drugs) compared to the non-PIP group (9 drugs). They also reported a rate of 40.4% for polypharmacy and 50.9% for extreme polypharmacy [25], compared to 59.71% and 26.62% in our study. In a study conducted in Romania on prescriptions in ambulatory elderly and institutionalized patients, Primejdie et al., reported a median number of 3, respectively 8 prescribed drugs [24]. The low number of drugs prescribed in ambulatory elderly can be explained by the fact that a maximum of 7 drugs can be prescribed on electronically reimbursed prescriptions in Romania [33]. 

Hypertension was the most common diagnosis, present in over 80% of our elderly patients. In line with our results, hypertension was the most frequent diagnosis in other studies conducted in different settings in Romania [31,34] and also in a similar study conducted in an internal medicine department in Spain [25]. 

Overall, the median number of comorbidities was 13.52 diagnoses, higher in patients with PIP (14.36) versus patients with non-PIP (12.48), while 58.51% of patients had a moderate CCI and 21.82% a severe CCI. Nam et al. in a study performed in Korean older adults, reported lower prevalence of patients with moderate CCI (40.14%) and similar for patients with severe CCI (20.46%) compared to our results [11]. We found a CCI median value of 3 across all groups, statistically significant between PIP and non-PIP groups, while a median value of 2 was reported in a study conducted in Spain [35], both values being in the moderate range. 

### 3.2. PIP Prevalence and Analysis 

The present study showed that 55.16% of the patients included had at least one PIP. Other studies conducted in internal medicine departments using STOPP-2 criteria have reported lower (41.5%) [25] or similar (51.4%) [22] rates to our findings. Compared to studies conducted in Romania, our results are higher than the prevalence (25.80%) of PIMs reported by Buda et al., when applying STOPP-2 criteria on ambulatory prescriptions [12], and lower than the PIMs (82.41%) in institutionalized patients reported by Primejdie et al., using STOPP-1 and PRISCUS list [24]. Different prevalence of PIM can be expected given the different settings of these studies, taking into account that ambulatory patients might not be as ill as the institutionalized ones, the latest being more prone to polypharmacy, or even the fact that the electronic prescriptions for chronic conditions in ambulatory older adults, evaluated at the pharmacy level, might not contain all drugs taken by the patient (e.g., non-prescription drugs, non-steroidal antiinflammatory drugs (NSAID), aspirin, antihistamines). Moreover, evaluating pharmacy level electronic prescriptions data does not allow evaluation of disease history and patients’ risk factors. Therefore, several STOPP criteria cannot be applied, hence the lower PIP prevalence in ambulatory patients. On the other side, the high prevalence of PIP in institutionalized older adults can be explained by the fact that institutionalization itself is a risk factor for PIP [36].

The most prevalent PIP was the prescription of aspirin, clopidogrel, dipyridamole, vitamin K antagonists, direct thrombin inhibitors or factor Xa inhibitors with concurrent significant bleeding risk (i.e., severe uncontrolled hypertension, bleeding diathesis, recent non-trivial spontaneous bleeding), found in 23.98% patients and accounted for 29.50% of PIPs found, similar with other study results where this PIP was prevalent (19.4%) [22]. This may be explained by the large representation of patients with hypertension in our study and by having the ATC drug class B01 as the most prescribed in these patients. There are several studies reporting a bleeding risk associated with the use of antithrombotic agents in patients with uncontrolled blood pressure [37,38,39]. Although a risk, the European Society of Cardiology Guideline recommends antiplatelet agents for secondary prevention in hypertensive patients, as they have proven to reduce the cardiovascular risk by ~4% in hypertensive patients [10]. Therefore, careful benefit-risk evaluation is needed in each individual elderly patient, as some might benefit from antiplatelet therapy without any harm.

The prescription of aldosterone antagonists with concurrent potassium-conserving drugs without recommendations for serum potassium monitoring was also found in 7.67% of patients, accounting for 8.36% of PIPs. Buda et al., found this association in only 0.15% of patients in their study conducted in an ambulatory setting [12]. Although in practice combinations of ACEi or angiotensin receptor blockers (ARBs) with aldosterone antagonists can be found, as the addition of aldosterone antagonists to the existing treatments is recommended for the management of resistant hypertension [10], or for preventing complications associated with chronic kidney disease [40], there is an associated risk of hyperkalemia. Therefore, regular serum potassium monitoring is highly recommended whenever this combination is beneficial for the patient, especially in older adults. 

In elderly patients with pre-existing hyperkalemia, there is a risk of kidney impairment and worsening/severe hyperkalemia when prescribed ACEi or ARBs, such prescribing being found in 3.84% of patients and accounting for 4.44% of PIPs, including dual renin-angiotensin system blockade by associating an ACEi with an ARB in one patient. Buda et al. reported co-prescription of ACEi with ARBs in 2.33% of ambulatory patients [31]. Although with low prescription rates, the combination of drugs acting on the renin-angiotensin system is still present in Romanian clinical practice, despite the clear recommendations on avoiding this association issued at the European level in 2014, except the situation when dual blockade is considered absolutely necessary and when it must be carried out under specialist supervision with close monitoring of kidney function, fluid and salt balance [41]. 

The prescription of NSAIDs along with vitamin K antagonists, direct thrombin inhibitors or factor Xa inhibitors was reported in 20 (4.80%) patients. In 18 (4.32%) patients, NSAIDs were prescribed despite a diagnosis of severe hypertension or severe heart failure, while eight (1.92%) patients with renal failure had prescribed NSAIDs. Bradley et al., in their analysis using START -I criteria, found in only 0.2% of patients the association of warfarin and NSAIDs, in 0.04% the prescription of NSAIDs with heart failure and in 0.1% the prescription of NSAID with chronic renal failure [26]. In Romania, Buda et al., also reported lower prevalence of this PIP, with 1.33% of patients having prescribed cyclooxygenase-2 (COX2) selective NSAIDs with cardiovascular events and 0.43% of patients having prescribed vitamin K antagonists with NSAIDs [31]. The increased prevalence of NSAIDs prescribed with cardiac therapy or cardiac disease compared to other studies can be explained by the fact that we conducted the study in an internal medicine setting, where patients with cardiovascular pathology are more prevalent. However, we did not find the prescription of COX2 selective NSAIDs with cardiovascular events. Caution is necessary with the use of NSAIDs in these elderly patients, as it can be associated with further cardiovascular adverse events such as increased blood pressure or congestive heart failure, aside from nephrotoxicity and gastrointestinal toxicity. Therefore, a benefit-risk evaluation is recommended for each patient [30].

### 3.3. Risk Factors for PIP

Several risk factors have been identified and reported in literature for PIP, such as physiologic changes, number of prescribed drugs, age [12] or female gender [27]. A link between PIP and specific diseases—such as chronic kidney disease [25] or the severity of the diseases based on CCI was also reported [11]. 

Our results indicated polypharmacy (OR: 2.908, *p* = 0.002) and extreme polypharmacy (OR: 5.236, *p* < 0.001) as independent risk factors for PIP. These results are in accordance with other findings, given that polypharmacy [12,25,26,31,42] and extreme polypharmacy [25] was reported in several studies as a risk factor for PIP. Polypharmacy is well known to be prevalent in older adults and is usually related to the number of comorbidities. However, polypharmacy is also related to reduced adherence to treatment and with increased risk of ADRs, hospitalizations and mortality [5,43]. Therefore, it is important to consider medication safety in polypharmacy.

We have also found essential hypertension (OR: 3.238, *p* < 0.001) and congestive heart failure (OR: 1.920, *p* = 0.010) as independent factors for PIP. This is expected considering that hypertension was the most frequent diagnosis and the PIP related to cardiac disease is the most prevalent as well in our study group. No other studies have reported these two diseases as being independent risk factors for PIP. Given that hypertension has an increased prevalence among patients in Romania and cardiac diseases are a leading cause of death [3], a review of the prescribed drugs using STOPP-2 criteria is recommended in these patients group.

Other studies have reported the severity of the disease based on CCI as an independent predictor for PIP [11,44]. Similarly, we found a positive correlation between PIP and an increased CCI. However, we did not find it as an independent risk factor for PIP. 

Female gender represented a higher percentage of the Overall group (61.63%), with a slightly higher representation in the PIP group as compared to the non-PIP group. Although there was a positive association between the female gender with PIP, we did not find the female gender to be an independent risk factor for PIP. Increased prevalence for the female gender was also reported in other studies evaluating PIP [12,22,24,29], however it is not clear how the female gender is a risk factor. Females generally have a better representation in studies compared to men [12,25,26,27,28] and have increased longevity [2]. Age was found to be a risk factor in some studies [12], while others did not find this association [25,31,42]. We also did not find age to be an independent risk factor for PIP.

Methods such as following prescribing protocols and guidelines [18], medication review [45] and reconciliation [46] are a good way to improve prescribing appropriateness. Medication review can be done by applying implicit or explicit criteria, which have proven to be a good interventional solution to reduce PIP [19,45]. In practice, most often it is recommended to use explicit criteria, although sometimes it is difficult due to the long list of criteria and medicine recommended for review [19]. The application of STOPP-2 criteria is a useful tool for PIP reduction, compared to usual pharmaceutical care [16]. Clinical pharmacists’ intervention to perform medication reconciliation and apply medication review criteria is a good way to reduce PIP [46,47].

### 3.4. Strengths and Limitations

To our knowledge, this is the first study conducted in Romania at hospital discharge, evaluating discharge prescriptions. Other studies conducted in Romania [12,24,31,34] so far have been performed in institutionalized patients and in an ambulatory setting (evaluating electronic prescriptions in a pharmacy setting). During hospitalization patients can undergo different changes in therapy, and at discharge, 2 out of 5 patients have discrepancies (e.g., duplications, omissions or inappropriate medication) in their medication [32]. A review of the prescribed medicines at the moment of discharge can be key for the timely identification of PIP and therapy adjustments. In our study, we have analyzed PIP for all the medicines that the patient was prescribed, regardless of the duration of treatment. Moreover, we had access to medical charts from the hospitalization period, therefore data such as medical history and laboratory values were available for evaluation of the specific STOPP criteria. Such data is not available in electronic prescribing at pharmacy level. 

Our study has several limitations such as manual analysis of discharge papers and data transfer into a database for further analysis, which can be prone to errors. However, the limitation was reduced by data analysis performed by two clinical pharmacists and comparing findings, with a third pharmacist consulted in case of analysis differences, aside from performing data quality checks in the database. Another limitation encountered in our study was due to the limited availability or unavailability of certain data (e.g., full medical history, treatment duration) in the discharge papers. This has led to not being able to evaluate all STOPP-2 criteria, therefore a specific number of STOPP-2 was selected and applied. Certain laboratory analyses were also not available in the discharge papers. Thus, we could not apply certain STOPP criteria that required specific laboratory test results. The study was conducted in a single hospital and a single region; therefore, data cannot be generalized. Moreover, it has been conducted in an internal medicine department, where only specific pathologies and associated therapies are available; therefore, STOPP criteria for drugs acting on the respiratory or nervous system could not be applied. Nevertheless, internal medicine departments are covering most pathologies. Although the necessary data was available for a relevant analysis of discharge prescriptions, a full overview of the entire treatment of the patients (e.g., treatment prescribed by other specialists or OTCs medication) was not available, which could lead to underestimation of polypharmacy. Furthermore, the available data did not allow the evaluation of ADRs linked to PIP. Further researches are needed to better understand this aspect.

## 4. Materials and Methods

### 4.1. Study Design, Setting and Participants

We conducted a retrospective observational cross-sectional study on patients discharged from a 100 beds Internal Medicine Department at a tertiary university hospital in North Western Romania. The study included male and female patients aged ≥65 years discharged between 1 January and 30 June 2018, regardless of their hospitalization duration. 

Patients with several hospital admissions were considered once, only their latest admission being included. Patients with fatal outcome or transferred to other departments or hospitals and those with no drugs from the STOPP criteria were excluded from the analysis. 

Patients’ anonymity and data confidentiality were preserved in accordance with the Romanian personal data protection legislation. The study was approved by the Ethics Committee of “Iuliu Haţieganu” University of Medicine and Pharmacy no. 70/11.03.2019. 

### 4.2. Data Collection and Variables

All eligible patient discharge medical charts were analyzed independently by two clinical pharmacists and their findings were compared. In case of differences in the results of the analysis, a third pharmacist was consulted and the results were discussed. 

Data analyzed in the study included: sex, age, discharge diagnoses (International Classification of Disease-ICD10 coding) and outcome, number of hospitalization days and prescribed medication at discharge. Relevant blood test results for the analysis were also collected, and the most recent test results were considered in case of repeated tests. We also evaluated chronic conditions and history of other relevant medical conditions for applying STOPP criteria.

Polypharmacy was defined as the prescription of ≥5 drugs, while the prescription of more than 10 drugs was defined as extreme polypharmacy [26]. We counted all drugs prescribed at discharge; however, polypharmacy was evaluated in terms of the number of prescribed drugs with a duration of prescription over 30 days, excluding dietary supplements and topical medications. All prescribed medicines were coded using the ATC classification system. 

CCI was calculated based on ICD10 coding using an Excel based calculator [21]. The CCI was further used to evaluate the potential impact of comorbidities on PIP [11,26,44]. 

### 4.3. Outcomes

The main outcome of this evaluation was the prevalence of PIP in the discharge prescriptions according STOPP-2 [15]. The secondary outcome of the analysis was the characterization of PIP, including the most common drugs involved in PIP, and their associated risk factors. For the expected prevalence of 41.5% in an internal medicine department [25], the required sample size was 374 for the margin of error or absolute precision of ±5% in estimating the prevalence with 95% confidence. With this sample size, the anticipated 95% CI was (36.5%, 46.5%). This sample size was calculated using a sample size calculator [48]. 

STOPP-2 comprises a list of 80 explicit criteria that help prescription review, focusing also on the patients’ clinical status [15]. Based on the data available, some criteria could not be applied or were difficult to correctly assess, therefore we applied a 34 criteria subset for PIP evaluation [9,11,37,49]. The criteria were not considered fulfilled unless all information was present in the charts (e.g., if a patient was prescribed a beta blocker and had a diagnosis of heart failure, without the NYHA classification mentioned, criteria B2 was not considered fulfilled). Similarly, for 66 discharge papers certain laboratory tests were missing, thus if for example, a patient was prescribed an AINS and the estimated glomerular filtration rate (eGFR) was not available, we did not consider the criteria E4 fulfilled. The full list of criteria applied in the present study is available in Appendix A. 

### 4.4. Statistical Analysis 

The numerical variables were expressed using medians and interquartile ranges (IQR) for non-normal distributed variables or means and standard deviation for normal distributed variables. Categorical data were expressed using numbers and percentages. For the comparison of patients with and without PIP we performed the Student’s *t*-test for normal distributed numerical variables, Mann-Whitneys’ test for non-normally distributed numerical variables. Chi-square test was used for categorical variables to determine the association with PIP. 

Logistic regression models were used to assess the presence of PIP for each variable. The independent effects of the different covariates on the presence/absence of PIP were studied by fitting a multivariate logistic regression model and estimating the odds ratio for each study variable controlling for covariables that were statistically significant in the crude models. We entered into the multivariate model age, gender and hospitalization as possible cofounders even though they were not statistically significant in the univariate analysis. The number of diagnoses was removed from the model due to its multicollinearity with CCI score (high variance inflation factor). The assumption of linearity to the logic was checked with a general additive model with splines for each continuous variable. The assumption hold for all continuous variables except for the number of drugs that was replaced in a model with a categorical one (polypharmacy: 1–4, 5–9, ≥10). IBM SPSS Statistics v23.0.0.0. and R environment for statistical computing and graphics v4.1.2 were used for statistical analysis. *p* < 0.05 was considered significant.

## 5. Conclusions

More than half of patients had at least one PIP at discharge. Patients with hypertension or cardiac disease and the ones with polymedication were prone to PIP. Antithrombotic drugs, agents acting on the renin-angiotensin system, aside from benzodiazepines and NSAIDs were most frequently involved in PIP. Cautious consideration of these PIPs in older adults is needed, as PIP might be associated with ADRs and subsequent hospitalization, prolonged hospitalization and additional costs. In clinical practice, tools such as STOPP criteria, which are very useful for monitoring prescriptions and identifying PIMs in older patients with comorbidities and polypharmacy.

## Figures and Tables

**Table 1:** General characteristics of the study group.

Variable	Overall Group (N = 417)	PIP (N = 230)	Non-PIP (N = 187)	*p*-Value
Age, years (mean ± SD)	75.62 ± 7.07	75.76 ± 6.96	75.45 ± 7.21	0.655
Gender (female, n [%])	257 (61.63)	148 (64.35)	109 (58.29)	0.206
State as discharge (improved, n [%])	403 (96.64)	225 (97.83)	178 (95.19)	0.137
Hospitalization days (median, [IQR])	7 (5–10)	8 (5–10)	7 (5–9)	0.060
Hospitalization days (n [%])				0.349
*≤7*	220 (52.76)	114 (49.57)	106 (56.68)	
*8–14*	161 (38.61)	95 (41.30)	66 (35.29)	
*>14*	36 (8.63)	21 (9.13)	15 (8.02)	
CCI (median, [IQR])	3 (2–4)	3 (2–5)	3 (1–4)	0.006
CCI (n [%])				0.003
*0–1*	82 (19.66)	34 (14.78)	48 (25.67)	
*Moderate CCI (2–4)*	244 (58.51)	135 (58.70)	109 (58.29)	
*Severe CCI (≥5)*	91 (21.82)	61 (26.52)	30 (16.04)	
Polypharmacy (median, [IQR])	*8 (6–10)*	8.5 (7–10)	6 (5–9)	<0.001
Polypharmacy (n [%])				<0.001
*≥5 drugs*	360 (86.33)	215 (93.48)	145 (77.54)	
*Polypharmacy (5–9 drugs)*	249 (59.71)	135 (58.70)	114 (60.96)	
*Extreme polypharmacy (≥10 drugs)*	111 (26.62)	80 (34.78)	31 (16.58)	
10 most common prescribed drugs (n [%]) *				
*Antithrombotic agents (B01)*	313 (75.06)	190 (82.61)	123 (67.21)	<0.001
*Beta blocking agents (C07)*	276 (66.19)	156 (67.83)	120 (65.57)	0.433
*Lipid modifying agents (C10)*	222 (53.24)	134 (58.26)	88 (48.09)	0.023
*Agents acting on the renin–angiotensin system (C09)*	215 (51.56)	137 (59.57)	78 (42.62)	<0.001
*Diuretics (C03)*	195 (46.76)	122 (53.04)	73 (39.89)	0.004
*Cardiac therapy (C01)*	188 (45.08)	116 (50.43	72 (39.34)	0.015
*Calcium channel blockers (C08)*	166 (39.81)	105 (45.65)	61 (33.33)	0.007
*Drugs for acid related disorders (A02)*	142 (34.05)	70 (30.43)	72 (39.34)	0.084
*Analgesics (N02)*	130 (31.18)	81 (35.22)	49 (26.78)	0.048
*Drugs used in diabetes (A10)*	97 (23.26)	65 (28.26)	32 (17.49)	0.007
Number of diagnoses at discharge (mean ± SD)	13.52 ± 4.33	14.36 ± 4.18	12.48 ± 4.30	<0.001
10 most common diagnoses at discharge (n [%])				
*Essential (primary) hypertension (I10)*	343 (82.25)	208 (90.43)	135 (72.19)	<0.001
*Mitral (valve) insufficiency (I34.0)*	217 (52.04)	123 (53.48)	94 (50.27)	0.514
*Fatty (change of) liver, not elsewhere classified (K76.0)*	154 (36.93)	84 (36.52)	70 (37.43)	0.848
*Atrial fibrillation and flutter (I48.0)*	157 (37.65)	101(43.91)	56 (29.95)	0.003
*Congestive heart failure (I50.0)*	123 (29.50)	84 (36.52)	39 (20.86)	<0.001
*Chronic ischemic heart disease, unspecified (I25.9)*	108 (25.90)	59 (25.65)	49 (26.20)	0.898
*Tricuspid insufficiency (I07.1)*	109 (26.14)	58 (25.22)	51 (27.27)	0.635
*Left ventricular failure (I50.1)*	108 (25.90)	58 (25.22)	50 (26.74)	0.724
*Aortic (valve) insufficiency (I35.1)*	108 (25.90)	63 (27.39)	45 (24.06)	0.441
*Obesity due to excess calories (E66.0)*	105 (25.18)	66 (28.70)	39 (20.86)	0.067

CCI, Charlson Comorbidity Index; IQR, interquartile range; N, number of patients; *p*-values for comparison tests of patients with and without PIP; PIP, potential inappropriate prescribing; SD, standard deviation. * Drug class was counted only once per patient if different drugs of the same class were prescribed per patient. The percentage represents for how many patients a drug class was prescribed to.

**Table 2:** Top 10 most frequently identified STOPP-2 criteria among the total number of PIPs and patients.

STOPP-2 Criteria		N (%) Out of PIPs	N (%) Out of Patients
C3	Antithrombotic agents with concurrent significant bleeding risk	113 (29.50)	100 (23.98)
K1	Benzodiazepines (sedative, may cause reduced sensorium, impair balance)	38 (9.92)	38 (9.11)
B12	Aldosterone antagonists with concurrent potassium-conserving drugs without monitoring of serum potassium	32 (8.36)	32 (7.67)
C10	NSAID and vitamin K antagonist, direct thrombin inhibitor or factor Xa inhibitors in combination	20 (5.22)	20 (4.80)
C5	Aspirin in combination with vitamin K antagonist, direct thrombin inhibitor or factor Xa inhibitors in patients with chronic atrial fibrillation	19 (4.96)	19 (4.56)
L2	Use of regular (as distinct from PRN) opioids without concomitant laxative	18 (4.70)	17 (4.08)
H2	NSAID with severe hypertension or severe heart failure	18 (4.70)	18 (4.32)
A3	Any duplicate drug class prescription	18 (4.70)	18 (4.32)
B11	ACEi or angiotensin receptor blockers in patients with hyperkalemia	17 (4.44)	16 (3.84)
J1	Sulfonylureas with a long duration of action with type 2 diabetes mellitus	16 (4.18)	16 (3.84)

ACEi, angiotensin-converting-enzyme inhibitors; N, number of prescription where the STOPP-2 criteria were found in; NSAID, non-steroidal antiinflammatory drugs; PIP, potential inappropriate prescribing; PRN, Pro re nata; STOPP-2, screening tool of older people’s prescribing.

**Table 3:** Variables associated with potential inappropriate prescribing.

Variable	*p*-Value	OR	95% CI	
			Lower	Upper
Age	0.436	0.988	0.958	1.019
Gender (female)	0.352	0.809	0.518	1.264
Hospitalization days	0.324	1.027	0.974	1.084
Charlson comorbidity index	0.605	1.032	0.917	1.161
Polypharmacy				
*5–9 drugs versus 1–4 drugs*	0.002	2.908	1.475	5.735
*≥10 drugs versus 1–4 drugs*	<0.001	5.236	2.403	11.409
Essential (primary) hypertension (I10)	<0.001	3.238	1.810	5.792
Congestive heart failure (I50.0)	0.010	1.920	1.166	3.161
Atrial fibrillation and flutter (I48.0)	0.067	1.538	0.970	2.440

CI, confidence interval; OR, Odds Ratio.

## Data Availability

Data is contained within the article.

## Appendix A

**Table A1:** STOPP-2 Criteria included in the analysis.

Code	Criteria	N (%) Out of PIPs	N (%) Out of Patients
**Section A: Indication of medication**			
A3	Any duplicate drug class prescription e.g., two concurrent NSAIDs, SSRIs, loop diuretics, ACEi or anticoagulants (optimization of monotherapy within a single drug class should be observed prior to considering a new agent).	18 (4.70)	18 (4.32)
**Section B: Cardiovascular System**			
B1	Digoxin for heart failure with normal systolic ventricular function (no clear evidence of benefit)	9 (2.35)	9 (2.16)
B2	Verapamil or diltiazem with NYHA Class III or IV heart failure (may worsen heart failure).	6 (1.57)	6 (1.44)
B3	Beta-blocker in combination with verapamil or diltiazem (risk of heart block).	1 (0.26)	1 (0.24)
B4	Beta-blocker with bradycardia (<50/min), type II heart block or complete heart block (risk of complete heart block, asystole).	2 (0.52)	2 (0.48)
B9	Loop diuretic for treatment of hypertension with concurrent urinary incontinence (may exacerbate incontinence).	5 (1.31)	5 (1.20)
B11	ACEi or ARB in patients with hyperkalemia.	17 (4.44)	16 (3.84)
B12	Aldosterone antagonists (e.g., spironolactone, eplerenone) with concurrent potassium-conserving drugs (e.g., ACEI’s, ARB’s, amiloride, triamterene) without monitoring of serum potassium (risk of dangerous hyperkalemia i.e., >6.0 mmol/L—serum K should be monitored regularly, i.e., at least every 6 months).	32 (8.36)	32 (7.67)
**Section C: Antiplatelet/Anticoagulant Drugs**			
C3	Aspirin, clopidogrel, dipyridamole, vitamin K antagonists, direct thrombin inhibitors or factor Xa inhibitors with concurrent significant bleeding risk, i.e., uncontrolled severe hypertension, bleeding diathesis or recent non-trivial spontaneous bleeding (high risk of bleeding).	113 (29.50)	100 (23.98)
C5	Aspirin in combination with vitamin K antagonist, direct thrombin inhibitor or factor Xa inhibitors in patients with chronic atrial fibrillation (no added benefit from aspirin)	19 (4.96)	19 (4.56)
C6	Antiplatelet agents with vitamin K antagonist, direct thrombin inhibitor or factor Xa inhibitors in patients with stable coronary, cerebrovascular or peripheral arterial disease (no added benefit from dual therapy).	4 (1.04)	4 (0.96)
C10	NSAID and vitamin K antagonist, direct thrombin inhibitor or factor Xa inhibitors in combination (risk of major gastrointestinal bleeding).	20 (5.22)	20 (4.80)
C11	NSAID with concurrent antiplatelet agent(s) without PPI prophylaxis (increased risk of peptic ulcer disease)	-	-
**Section D: Central Nervous System and Psychotropic Drugs**			
D8	Anticholinergics/antimuscarinics in patients with delirium or dementia (risk of exacerbation of cognitive impairment).	2 (0.52)	2 (0.48)
D14	First-generation antihistamines (safer and less toxic antihistamines now widely available).	4 (1.04)	4 (0.96)
**Section E: Renal System**			
E1	Digoxin at a long-term dose greater than 125 µg/day if eGFR < 30 mL/min/1.73 m^2^ (risk of digoxin toxicity if plasma levels not measured).	1 (0.26)	1 (0.24)
E2	Direct thrombin inhibitors (e.g., dabigatran) if eGFR < 30 mL/min/1.73 m^2^ (risk of bleeding)	-	-
E3	Factor Xa inhibitors (e.g., rivaroxaban, apixaban) if eGFR < 15 mL/min/1.73 m^2^ (risk of bleeding)	-	-
E4	NSAID’s if eGFR < 50 mL/min/1.73 m^2^ (risk of deterioration in renal function).	8 (2.09)	8 (1.92)
E5	Colchicine if eGFR < 10 mL/min/1.73 m^2^ (risk of colchicine toxicity)	-	-
E6	Metformin if eGFR < 30 mL/min/1.73 m^2^ (risk of lactic acidosis)	3 (0.78)	3 (0.72)
**Section F: Gastrointestinal System**			
F4	Oral elemental iron doses greater than 200 mg daily (e.g., ferrous fumarate > 600 mg/day, ferrous sulphate > 600 mg/day, ferrous gluconate > 1800 mg/day; no evidence of enhanced iron absorption above these doses)	12 (3.13)	12 (2.88)
**Section G: Respiratory System**			
G1	Theophylline as monotherapy for COPD (safer, more effective alternative; risk of adverse effects due to narrow therapeutic index).	-	-
G2	Systemic corticosteroids instead of inhaled corticosteroids for maintenance therapy in moderate–severe COPD (unnecessary exposure to long-term side-effects of systemic corticosteroids and effective inhaled therapies are available)	-	-
G4	Benzodiazepines with acute or chronic respiratory failure i.e., pO_2_ < 8.0 kPa ± pCO_2_ > 6.5 kPa (risk of exacerbation of respiratory failure)	1 (0.26)	1 (0.24)
**Section H: Musculoskeletal System**			
H2	NSAID with severe hypertension (risk of exacerbation of hypertension) or severe heart failure (risk of exacerbation of heart failure)	18 (4.70)	18 (4.32)
H5	Corticosteroids (other than periodic intra-articular injections for mono-articular pain) for osteoarthritis (risk of systemic corticosteroid side-effects)	-	-
H7	COX-2 selective NSAIDs with concurrent cardiovascular disease (increased risk of myocardial infarction and stroke)	-	-
H8	NSAID with concurrent corticosteroids without PPI prophylaxis (increased risk of peptic ulcer disease)	-	-
H9	Oral bisphosphonates in patients with a current or recent history of upper gastrointestinal disease i.e., dysphagia, oesophagitis, gastritis, duodenitis, or peptic ulcer disease or upper gastrointestinal bleeding (risk of relapse/exacerbation of oesophagitis, oesophageal ulcer, oesophageal stricture)	3 (0.78)	3 (0.72)
**Section J. Endocrine System**			
J1	Sulfonylureas with a long duration of action (e.g., glibenclamide, chlorpropamide, glimepiride) with type 2 diabetes mellitus (risk of prolonged hypoglycaemia)	16 (4.18)	16 (3.84)
**Section K: Drugs that predictably increase the risk of falls in older people**			
K1	Benzodiazepines (sedative, may cause reduced sensorium, impair balance)	38 (9.92)	38 (9.11)
K4	Hypnotic Z-drugs (e.g., zopiclone, zolpidem, zaleplon) may cause protracted daytime sedation, ataxia	13 (3.39)	13 (3.12)
**Section L: Analgesic Drugs**			
L2	Use of regular (as distinct from PRN) opioids without concomitant laxative (risk of severe constipation)	18 (4.70)	17 (4.08)

ARB, angiotension receptor blockers; ACEi, angiotensin-converting enzyme inhibitors; COPD, chronic obstructive pulmonary disease; COX-2, cyclooxygenase-2 selective; eGFR, estimated glomerular filtration rate; N, number of prescription where STOPP-2 criteria was found; NSAID, non-steroidal anti-inflammatory drugs; NYHA, New York Heart Association Classification; PIP, potential inappropriate prescribing; PRN, pro re nata; PPI, proton pump inhibitor; SSRI, selective serotonin reuptake inhibitors.
